# Computerized monitoring of patient-reported speech and swallowing problems in head and neck cancer patients in clinical practice

**DOI:** 10.1007/s00520-012-1422-y

**Published:** 2012-03-07

**Authors:** Ingrid C. Cnossen, Remco de Bree, Rico N. P. M. Rinkel, Simone E. J. Eerenstein, Derek H. F. Rietveld, Patricia Doornaert, Jan Buter, Johannes A. Langendijk, C. René Leemans, Irma M. Verdonck-de Leeuw

**Affiliations:** 1 Department of Otolaryngology/Head and Neck Surgery, VU University Medical Center, PO Box 7057, 1007 MB Amsterdam, The Netherlands; 2 Department of Radiation Oncology, VU University Medical Center, Amsterdam, The Netherlands; 3 Department of Medical Oncology, VU University Medical Center, Amsterdam, The Netherlands; 4 Department of Radiation Oncology, University Medical Center Groningen/University of Groningen, Groningen, The Netherlands

**Keywords:** Head and neck cancer, E-health, Patient-reported speech and swallowing problems, Quality of life, Emotional well-being

## Abstract

**Purpose:**

The purpose of this study is to evaluate computerized monitoring of speech and swallowing outcomes and its impact on quality of life (QoL) and emotional well-being in head and neck cancer patients in an outpatient clinic.

**Methods:**

Sixty-seven patients, treated by single or multimodality treatment, completed the EORTC QLQ-C30 and QLQ-H&N35 questionnaires and the Hospital Anxiety and Depression Scale in an outpatient clinic, using a touch screen computer system (OncoQuest), at baseline (at time of diagnosis) and first follow-up (1 month after end of treatment).

**Results:**

Tumor sites included oral cavity (*n* = 12), oropharynx (*n* = 18), hypopharynx (*n* = 8), and larynx (*n* = 29). Tumor stage included carcinoma in situ (*n* = 3), stage I (*n* = 21), stage II (*n* = 7), stage III (*n* = 15), and IV (*n* = 21). No speech or swallowing problems at baseline or follow-up were noted in 23 % (speech) and 41 % (swallowing) of patients. Twenty-one percent (speech) and 19 % (swallowing) had problems at baseline and returned to normal scores at follow-up, while 16 % (speech) and 19 % (swallowing) had normal scores at baseline and developed problems at follow-up. Forty percent (speech) and 21 % (swallowing) had persistent problems from baseline to follow-up. At baseline, speech problems were significantly related to tumor site and emotional distress. At baseline and follow-up, swallowing problems were significantly related to QoL and emotional distress. At follow-up, speech problems were significantly related to QoL, emotional distress, and swallowing problems.

**Conclusions:**

Monitoring speech and swallowing problems through OncoQuest in an outpatient clinic is feasible. Many patients report speech and swallowing problems, negatively affecting their QoL and emotional well-being.

## Introduction

Head and neck cancer (HNC) patients often have to deal with speech and swallowing problems before or after treatment, negatively affecting health-related quality of life. HNC patients may experience discomfort and functional deficits as a result of the disease itself, such as tumor-induced pain, and/or problems with swallowing and speaking prior to treatment. It has been estimated that 34–75 % of HNC patients have speech or swallowing problems after treatment [1–3]. In clinical practice, various subjective and objective measures to evaluate posttreatment speech and swallowing outcomes are used. Speech outcomes can be assessed by indicators of speech production (e.g., oral function and articulation tests and aerodynamic and acoustical analyses), perceptual speech evaluation (e.g., intelligibility, articulation, nasality, speech rate, and acceptability), and by subjective measures to evaluate self-reported speech problems in everyday life situations (e.g., questionnaires). Swallowing outcome measures include objective assessment methods such as the modified barium swallow procedure with videofluorography [4], videofluoroscopy combined with manometry (manofluoroscopy) [5], fiberoptic endoscopic examination of swallowing [6], or scintigraphy [7]. Other clinician-rated dysphagia assessments can be performed by clinical swallowing evaluation (e.g., gathering information on current swallowing problems, reviewing medical history, observing signs relevant to the patient's medical status, conducting an examination of speech and swallowing structures, observing the patient during trial swallows, and by recording acute and late toxicity after radiotherapy) [8].

Patient-reported speech and swallowing problems are usually identified through questionnaires. Implementation of patient-reported outcomes in clinical practice may be facilitated by information technology, allowing real-time quick and easy presentation of results to clinicians [9–11]. In our institute, a touch screen computer-assisted data collection system, OncoQuest, was developed and implemented in clinical practice enabling structured monitoring of quality of life and emotional distress [12, 13].

The purpose of this explorative study is to evaluate structured computerized monitoring of prospective patient-reported speech and swallowing outcomes from baseline (pretreatment) to first follow-up 1 month after treatment in HNC patients using OncoQuest and to investigate the impact of speech and swallowing problems on quality of life and emotional well-being.

## Methods

### Patients

From February 2009 to July 2010, 67 newly diagnosed HNC patients filled out the patient-reported outcomes through OncoQuest. The inclusion criteria were curative treatment for primary tumors in the larynx, hypopharynx, oral cavity, or oropharynx. Exclusion criteria were: diseases causing cognitive dysfunction and poor understanding of the Dutch language. Age, gender, tumor site and stage, and treatment modality were recorded. Informed consent was obtained from all patients. This study was approved by the VU University medical ethics committee.

### Outcome measures

**Fig. 1:**
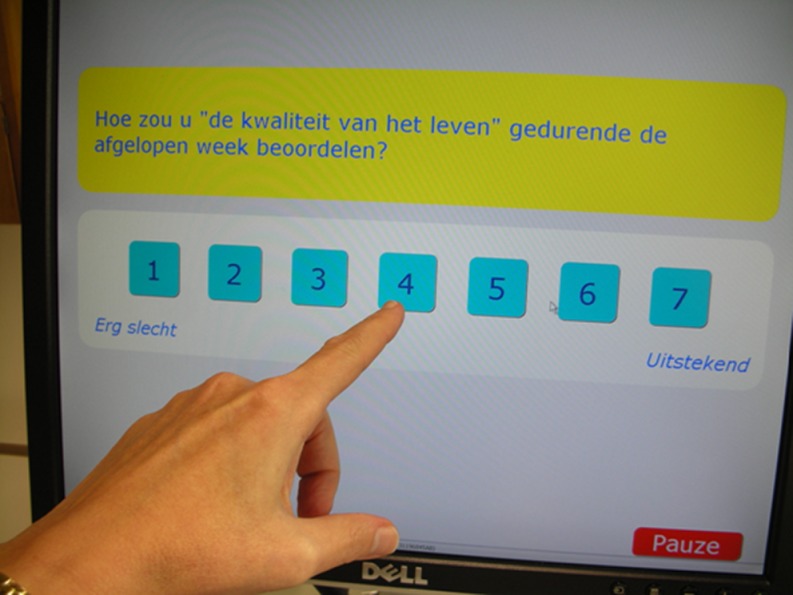
Example of the user interface of OncoQuest

Patients completed the EORTC QLQ-C30 [14] and EORTC QLQ-H&N35 [15] and the Hospital Anxiety and Depression Scale (HADS) [16] at the time of diagnosis and on their first follow-up after the end of treatment. The questionnaires were presented through OncoQuest, a touch screen computer-based data collection system. An example of the touch screen user interface is shown in Fig. 1. One of the outcome variables in OncoQuest is time to complete the questionnaire: at their first visit, it took patients on average 8.7 min to complete all 79 items, and 8.0 min at first follow-up visit, 1 month after treatment.

The EORTC QLQ-C30 is a cancer-specific questionnaire and comprises a global health-related quality of life (HRQOL) scale (two items) and five functional scales: physical functioning (five items), role functioning (two items), emotional functioning (four items), cognitive functioning (two items), and social functioning (two items). There are three symptom scales (fatigue (three items), nausea and vomiting (two items), and pain (two items)) and six single items relating to dyspnoea, insomnia, loss of appetite, constipation, diarrhea, and financial difficulties. The QLQ-C30 subscale Global QOL is used in the present study.

The EORTC QLQ-H&N35 module covers specific HNC issues and comprises seven subscales: pain (four items), swallowing (five items), senses (two items), speech (three items), social eating (four items), social contact (five items), and sexuality (two items). There are ten single items covering problems with teeth, dry mouth, sticky saliva, cough, opening the mouth wide, weight loss, weight gain, use of nutritional supplements, feeding tubes, and pain-killers. The speech and swallowing subscales were used in the present study.

The scores of the QLQ-C30 and of the QLQ-H&N35 are linearly transformed to a scale of 0 to 100, with a higher score indicating a higher (i.e., more positive) level of functioning or global HRQOL, or a higher (i.e., more negative) level of symptoms or (speech and swallowing) problems. Presence of speech or swallowing problems was defined as a score ≥10 on the EORTC QLQ-H&N35 speech or swallowing subscale. These cutoff scores are based on a cohort of 110 subjects from the general population of whom 95 % scored below 10 on these two subscales (unpublished data).

The HADS is a 14-item scale with two subscales, anxiety and depression. The total HADS score ranges from 0 to 42; the subscales range from 0 to 21. In psycho-oncology, the total HADS score is proven to be an accurate instrument to identify cancer patients with depression [17, 18] and anxiety and other psychological sequelae. In the present study, a total HADS score of >15 was used as indicator of a high level of distress [19, 20].

### Statistical analysis

Patients were categorized regarding presence of speech problems (yes/no) or swallowing problems (yes/no), gender, tumor site (larynx/hypopharynx/oropharynx/oral cavity), tumor stage (carcinoma in situ/I/II/III/IV), and treatment: surgery/radiotherapy/chemoradiation/surgery (other than laryngectomy) and radiotherapy/surgery (laryngectomy) and radiotherapy/surgery and chemoradiation. Chi-square tests were used to investigate the relation between the presence of speech or swallowing problems and gender, tumor site (larynx/hypopharynx vs. oral/oropharynx), tumor stage (cis/I/II vs. III/IV), and treatment modality: single modality (surgery or radiotherapy) vs. combined modality (surgery and radiotherapy/chemoradiation). Pearson correlation coefficients were used to test associations between speech or swallowing problems and global QOL (global QOL scale EORTC QLQ-C30) and emotional distress (total HADS score). For all tests, a *p* value less than .05 was considered statistically significant.

## Results

### Patients

The patient group consisted of 51 males (76 %) and 16 females (24 %). Mean age was 64 years (range 43–83). Tumor sites included oral cavity (*n* = 12), oropharynx (*n* = 18), hypopharynx (*n* = 8), and larynx (*n* = 29). Tumor stage included carcinoma in situ (*n* = 3), stage I (*n* = 21), stage II (*n* = 7), stage III (*n* = 15), and IV (*n* = 21). Patients were treated by surgery (*n* = 18), radiotherapy (*n* = 23), chemoradiation (*n* = 12), surgery (other than laryngectomy) and postoperative radiotherapy (*n* = 7), laryngectomy and radiotherapy (*n* = 6), and surgery and postoperative chemoradiation (*n* = 1; Table 1). Regarding assessment at first follow-up visit (follow-up), median time since the end of treatment was 1 month (SD 1.2 months).

**Table 1:** Characteristics of 67 patients

	*N* (%)
Gender	
Male	51 (76%)
Female	16 (24%)
Mean age in years	64 (9.6, 43–83) (SD, range)
Tumor site	
Oral cavity	12 (18%)
Oropharynx	18 (27%)
Hypopharynx	8 (12%)
Larynx	29 (43%)
T classification (stage)	
Carcinoma in situ	3 (5%)
I	21 (31%)
II	7 (11%)
III	15 (22%)
IV	21 (31%)
Treatment	
Surgery	18 (27%)
Radiotherapy	23 (34%)
Chemoradiation	12 (18%)
Surgery and radiotherapy(other than laryngectomy)	7 (10%)
Surgery and radiotherapy (laryngectomy)	6 (9%)
Surgery and chemoradiation (other than laryngectomy)	1 (2%)

### Patient-reported speech outcome

**Fig. 2:**
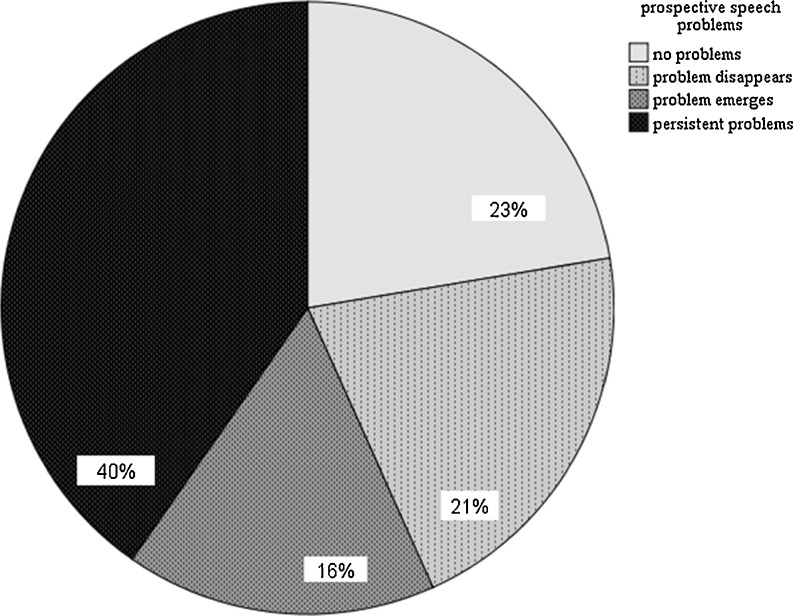
Prospective speech problems (*n* = 67)

Mean EORTC QLQ-H&N35 speech subscale score was 22.72 (SD 26.3; range 0–100) at time of diagnosis and 29.52 (SD 25.5; range 0–89) at follow-up (Table 2). No patient-reported speech problems at baseline or follow-up were noted in 23 % of the patients, 21 % had speech problems at baseline and returned to normal scores at follow-up, while 16 % of the patients had normal scores at baseline and developed problems at follow-up. Forty percent of the patients had persistent problems from baseline to follow-up (Fig. 2). Speech reported outcomes were not significantly related to gender, tumor stage, or treatment modality. At baseline, speech problems were significantly related to tumor site (*χ* ^2^ = 10.28, *p* = .00; more speech problems in oral and oropharyngeal cancer compared to laryngeal/hypopharyngeal cancer). At time of diagnosis (baseline), patient-reported speech outcomes were significantly related to emotional distress (*r* = .25, *p* = .04). At follow-up, patient-reported speech outcomes were significantly related to quality of life (*r* = −.49, *p* = .00), emotional distress (*r* = .53, *p* = .00), and swallowing outcomes (*r* = .40, *p* = .00).

**Table 2:** Overview of mean scores and standard deviation (SD) on the EORTC QLQ-C30 (global quality of life), EORTC QLQ-H&N35 (speech problems), EORTC QLQ-H&N35 (swallowing problems), and HADS (distress) at baseline (at time of diagnosis) and first follow-up (1 month after end of treatment)

	Time of diagnosis		First follow-up visit	
	Mean	SD	Mean	SD
EORTC QLQ-C30 (global quality of life)	68.03	22.12	69.40	19.54
EORTC QLQ-H&N35 (speech problems)	22.72	26.27	29.52	25.54
EORTC QLQ-H&N35 (swallowing problems)	19.03	25.16	26.87	29.19
HADS (distress)	9.82	8.26	8.97	6.98

A higher mean score indicates a higher (i.e., more positive) level of functioning or global QOL. A higher mean score indicates a higher (i.e., more negative) level of speech, swallowing problems, or distress symptoms

### Patient-reported swallowing outcome

**Fig. 3:**
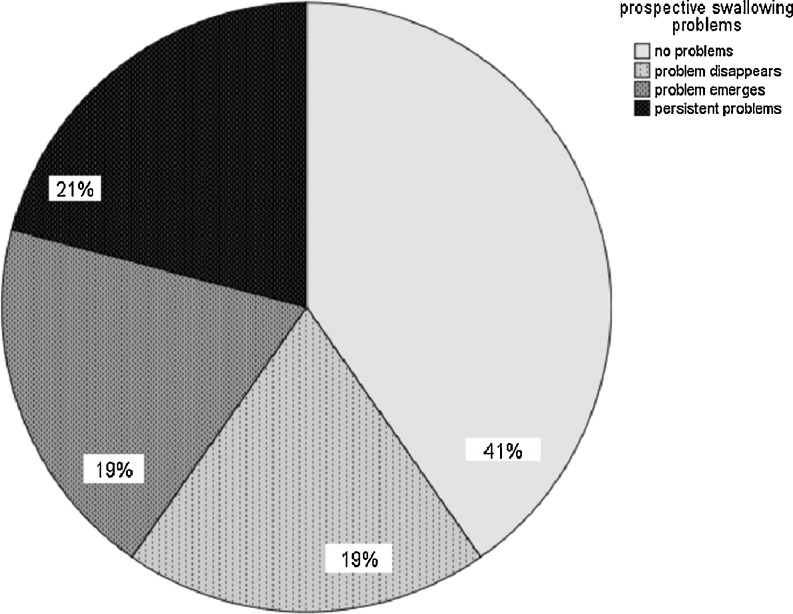
Prospective swallowing problems (*n* = 67)

At time of diagnosis, mean EORTC QLQ-H&N35 swallowing subscale score was 19.03 (SD 25.2; range 0–83) and at follow-up 26.87 (SD 29.2; range 0–92; Table 2). No swallowing problems at baseline or follow-up were noted in 41 % of the patients. Nineteen percent had swallowing problems at baseline and returned to normal scores at follow-up, while 19 % had normal scores at baseline and developed swallowing problems at follow-up; 21 % had persistent problems from baseline to follow-up (Fig. 3). Patient-reported swallowing outcomes were significantly related to quality of life at the time of diagnosis (*r* = −.51, *p* = .00) and at follow-up (*r* = −.54, *p* = .00), to emotional distress at time of diagnosis (*r* = .52, *p* = .00) and at follow-up (*r* = .46, *p* = .00), and to speech outcomes at follow-up (*r* = .40, *p* = .00). Swallowing reported outcomes were not significantly related to gender, tumor site and stage, or treatment modality.

## Discussion

This explorative study evaluated computerized monitoring of prospective patient-reported speech and swallowing outcomes in an outpatient clinic through OncoQuest, a touch screen computer system. A minority of the patients reported speech (21 %) or swallowing problems (19 %) at time of diagnosis, which is consistent with earlier studies [21, 22]. In the present study, the majority either developed speech or swallowing problems after treatment (16 and 19 %, respectively) or had persistent speech or swallowing problems from baseline to follow-up (40 and 21 %, respectively). In total, 56 % of the HNC patients had speech problems and 40 % had swallowing problems at first follow-up.

Previous studies of objective and subjective speech and swallowing problems yielded similar results regarding speech problems (ranging from 46 to 64 %) [23, 24], and swallowing problems (ranging from 30 to 75 %) [23, 25]. Although prevalence rates vary significantly due to methodological issues as inclusion criteria and assessment methods, it is clear that a substantial part of HNC patients report speech or swallowing problems before and/or after treatment with a clear impact on quality of life and emotional functioning. Also in earlier studies, speech and swallowing problems in HNC patients appeared to be significantly related to quality of life and emotional well-being [26–31], as shown in the present study. Structured monitoring of speech and swallowing problems and quality of life in clinical practice appears to be feasible, enhances patient–provider communication [32], and may facilitate physicians to focus quickly on issues that require further attention [10–12, 33] and to refer patients (if indicated) to speech and swallowing rehabilitation or other supportive care options in order to minimize acute and late effects of HNC and its treatment and to optimize quality of life.

In this study, two time points were chosen (before treatment and shortly after end of treatment) to obtain insight in acute side effects of treatment and possible needs for supportive care at an early stage. All patients completed the questionnaires at those time points. Longer follow-up will provide more information on the course of speech and swallowing problems. However, in clinical practice, it is clear that the willingness to complete the questionnaires drops over time. More long-term follow-up research is needed to assess efficacy of structured monitoring of speech and swallowing using a touch screen computer in clinical practice and to investigate moderating factors that may influence participation rate, such as age, gender, treatment modality, and burden of symptoms.

Speech and swallowing function can be improved in three ways. First, in planning surgery and (chemo-) radiation, head and neck oncologists may take into account the effects of their interventions on swallowing and speech production by using intensity-modulated radiotherapy to constrain the dose to be received by the swallowing muscles [34] and to minimize the impact on surrounding healthy tissues [35]. Second, a number of rehabilitative procedures are available to reduce or eliminate speech and swallowing problems after HNC surgery or (chemo-) radiation [36–38] by performing range of motion exercises, resistance exercises, swallow maneuvers, and compensation techniques. And third, speech and swallowing may be evaluated before treatment to determine the speech and swallowing status at start and to prepare the patient regarding possible speech and swallowing impairments.

It remains unclear whether patients might benefit from speech or swallowing rehabilitation (one of the main reasons to monitor patient-reported speech and swallowing outcome in the first place): efficacy studies of (pretreatment) speech and swallowing therapy are scarce [39–42]. A pilot study in our clinic revealed that mobility and flexibility exercises during a burdensome period of radiotherapy treatment are feasible. Exercises were easily learned and carried out according to plan. The protocol is extended with a DVD and a website (www.halszaken-vumc.nl) as helpful e-health tools with information, film clips with examples of the exercises, and e-coaching. A prospective study is ongoing to investigate the effectiveness of this exercise protocol during radiotherapy. Next to effectiveness, this study will also provide insight into determinants and barriers regarding participation and compliance.

In the present study, the speech and swallowing subscales of the EORTC QLQ-H&N35 module were used to assess patient-reported speech and swallowing problems because they comprise only three speech and five swallowing items, and are thus quick and easy to use in a busy outpatient clinic. We used a cutoff score of 10 based on a cohort of 110 subjects from the general population of whom 95 % scored below 10 on these two subscales (unpublished data). However, these short scales may not cover all speech- and swallowing-related issues. The Speech Handicap Index (SHI) and the Swallowing Questionnaire on Quality of Life (SWAL-QoL) may provide more specific information on self-reported speech and swallowing problems. The SHI was developed in a cohort of patients with oral or oropharyngeal cancer [43] and consists of 30 items on speech problems in daily life. The SHI was validated and a cutoff score of 6 (or higher) was defined on the total SHI scale to identify patients with speech problems in daily life after treatment for oral or oropharyngeal cancer, which was confirmed in a recent study on laryngeal cancer patients (unpublished data). In an earlier study, we translated and validated the 44-item swallowing-specific quality of life questionnaire SWAL-QoL and defined a cutoff score of 14 points (or higher) regarding the total SWAL-QOL score to identify patients with swallowing problems after treatment for oral or oropharyngeal cancer and [44]; this cutoff score was confirmed in a recent study on laryngeal cancer patients (unpublished data). Recently, based on the positive results of the present study regarding the feasibility to monitor speech and swallowing (as assessed by the short EORTC QLQ-H&N35 scales) in clinical practice, the SWAL-QOL and SHI have been built in OncoQuest. An ongoing research focuses on whether these longer questionnaires are also feasible in clinical practice.

## Conclusion

Computerized monitoring of patient-reported speech and swallowing outcome in a busy outpatient clinic using a touch screen computer system (OncoQuest) is feasible. Many HNC patients report speech and swallowing problems before and after treatment, negatively affecting QoL and emotional well-being.
